# The dynamic expression of SOX17 in germ cells from human female foetus and adult ovaries after specification

**DOI:** 10.3389/fendo.2023.1124143

**Published:** 2023-07-28

**Authors:** Ying-Yi Luo, Hui-Ying Jie, Ke-Jun Huang, Bing Cai, Xiu Zhou, Ming-Yi Liang, Can-Quan Zhou, Qing-Yun Mai

**Affiliations:** ^1^ Reproductive Medicine Center, The First Affiliated Hospital, Sun Yat-sen University, Guangzhou, China; ^2^ Reproductive Medicine Center, The First People’s Hospital of Foshan, Foshan, China; ^3^ Department of Obstetrics & Gynaecology, Guangzhou Women and Children’s Medical Center, Guangzhou, China

**Keywords:** SOX17, germ cells, VASA, mitosis, meiosis

## Abstract

**Background:**

SOX17 has been identified as a critical factor in specification of human primordial germ cells, but whether SOX17 regulates development of germ cells after sex differentiation is poorly understood.

**Methods:**

We collected specimens of gonadal ridge from an embryo (n=1), and ovaries of foetuses (n=23) and adults (n=3). Germ cells were labelled with SOX17, VASA (classic germ cells marker), phosphohistone H3 (PHH3, mitosis marker) and synaptonemal complex protein 3 (SCP3, meiosis marker).

**Results:**

SOX17 was detected in both cytoplasm and nucleus of oogonia and oocytes of primordial and primary follicles from 15 to 28 gestational weeks (GW). However, it was exclusively expressed in cytoplasm of oogonia at 7 GW, and in nucleus of oocytes in secondary follicles. Co-expression rates of SOX17 in VASA^+^ germ cells ranged from 81.29% to 97.81% in foetuses. Co-staining rates of SOX17 and PHH3 or SCP3 were 0%-34% and 0%-57%, respectively. Interestingly, we distinguished a subpopulation of SOX17^+^VASA^-^ germ cells in fetal ovaries. These cells clustered in the cortex and could be co-stained with the mitosis marker PHH3 but not the meiosis marker SCP3.

**Conclusions:**

The dynamic expression of SOX17 was detected in human female germ cells. We discovered a population of SOX17^+^ VASA^-^ germ cells clustering at the cortex of ovaries. We could not find a relationship between mitosis or meiosis and SOX17 or VASA staining in germ cells. Our findings provide insight into the potential role of SOX17 involving germ cells maturation after specification, although the mechanism is unclear and needs further investigation.

## 1 Introduction

Currently, the timeline of key events in human female germ cells is well-defined. Female germ cells, including primordial germ cells (PGCs), oogonia, and oocytes, play a critical role in genetic material transfer across a generation (1). Human PGCs first appear in the posterior epiblast of embryos around 4 gestational weeks (GW), although the origin of PGCs is still unclear. Approximately at 6 GW, human PGCs migrate to genital ridges and differentiate into oogonia with the interaction of gonadal somatic cells. After passing through leptotene, zygotene, and pachytene diplotene stages of meiosis prophase I, oogonia arrest at the dictyate stage to become primary oocytes. Then single-layer pre−granulosa cells surround primary oocytes to form primordial follicles. Primordial follicles further develop into primary, secondary and antral follicles during puberty accompanied by oocyte differentiation. Primordial follicles develop into primary follicles and secondary follicles through activation. After puberty, ovulated oocytes mature after completing meiosis. The process by which a primary oocyte completes the secondary meiotic division is termed oocyte nuclear maturation (2, 3).

Studies involving later stages of human germ cell development are difficult to carry out due to the limited availability of foetal ovarian tissue and ethical constraints. Various gene markers of germ cells at different developmental stages, such as OCT4, DAZL and VASA, have been described (4). Among these markers, VASA (also called DDX4), a member of the DEAD box family, was first identified to be critical in Drosophila oogenesis in 1988 and recognized as a relatively specific marker of human germ cells (5, 6). In addition, germ cells undergoing mitosis or meiosis can be labelled for phosphohistone H3 (PHH3) or synaptonemal complex protein 3 (SCP3). PPH3 is considered a classical mitosis marker. In mitotic cells, histone H3 is phosphorylated at serine 10 and serine 28. When serine 28 is phosphorylated, PHH3 is specifically detected (7). Synaptonemal complex, consisting of SCP1, SCP2 and SCP3, is critical to chromosome segregation during meiosis (8). Studies reported that SCP3 is expressed in human ovaries (9) and is widely used as a meiosis marker (10, 11).

Remarkable progress in inducing human PGCs *in vitro* has been made in recent years (12–15). In 2015, Irie et al. successfully developed human PGC-like cells (hPGCLCs) from differentiated embryonic stem cells (ESCs) and induced pluripotent stem cells (iPSCs). They first demonstrated the critical role of SOX17 in regulating hPGCLC specification (12). SOX17 belongs to the SRY-related box (SOX) family, located on human chromosome 8q11.23 (16). In 1996, two mRNA isoforms of the SOX17 gene were first isolated from the mouse testis cDNA library (17). Similar to other orthologous pairs of SOX genes, SOX17 is characterized by a high mobility group (HMG) box domain, which enables SOX17 to specifically combine with DNA (18). SOX17 is critical in the specification of mammalian embryo primitive endoderm (19) and regulates developmental processes in various organ systems (20–24). In addition, SOX17 might be a tumour suppressor of endometrial cancer, cholangiocarcinoma, colon carcinoma cells, and breast cancer by antagonizing the effect of the canonical Wnt/beta-catenin signalling pathway (19, 25–27).

SOX17 is pivotal for hPGCLC specification, although the molecular mechanisms are still not fully elucidated (28). Studies involving whether SOX17 regulates germ cells maturation after the specification period have rarely been reported. Herein, we describe the expression pattern of the SOX17 protein in different-stage germ cells in human foetal and adult ovaries and analyse the relationship between SOX17 expression and proliferation or first meiosis of human oogonia, which may shed some light on the oogenesis mechanism in human ovaries.

## 2 Materials and methods

### 2.1 Collection of human foetal ovaries and adult ovaries

This study was reviewed and approved by the Clinical Research Ethics Committee of the First Affiliated Hospital of Sun Yat-sen University (Ethical approval number (2016): 090) and carried out between 2016 and 2018. Women who participated in this study signed informed consent forms. We collected specimens of gonad ridge of an embryo (n=1), and ovaries of foetuses (n=23) from multiple pregnancy reduction surgery at 7 GW, inevitable spontaneous abortion or induced abortion because of severe malformation of foetus except for urogenital system. The malformations of foetus included cleft lip and palate, Down syndrome, severe thalassemia, ventricular dysplasia syndrome and Cantrell pentalogy. Gestational age was determined by ultrasonography (7 GW) and further confirmed by the foot size of the foetus (15–28 GW). In addition, three adults receiving ovariectomy due to autogenous diseases also gave consent for us to acquire their ovary specimens after surgical removal.

### 2.2 Histopathologic confirmation of human ovaries

Genital ridges or ovaries were fixed in 10% buffered formalin. After 24–48 hours of fixation, samples were processed for routine paraffin embedding. Embedded samples were serially cut into 4-μm-thick sections, mounted onto cleaned, coated slides and stored at room temperature until use. All samples were examined for tissue integrity and general histology using haematoxylin and eosin staining. These histological sections were all reviewed and confirmed by two pathologists.

### 2.3 Immunofluorescence

Multiplex immunofluorescence was performed on fixed sections of ovaries described above. Four-μm-thick sections of paraffin-embedded tissues were dried overnight, dewaxed, and rehydrated through xylene and a graded alcohol series [100% (1 min), 95% (1 min), 80% (1 min), and 70% (1 min)]. Antigen retrieval was performed by Pressure-cooking in citrate buffer (#AR0024, BOSTER, China) for 3 minutes, followed by incubation with 0.2% Triton X (#T8200, Solarbio, China) for 15 minutes. The sections were blocked in 5% donkey serum (#SL050, Solarbio) for 30 mins at room temperature and then incubated with primary antibodies (1:1) at 4 degrees centigrade for 12–14 hours. Primary antibody dilutions were as follows: SOX17 (1:20; #AF1924, R&D, USA), VASA (1:100; #AB27591, Abcam, USA), PHH3 (1:800; #AB47297, Abcam), and SCP3 (1:2000; #AB150292, Abcam). On Day 2, sections were incubated with secondary antibodies for 1 hour at room temperature. Secondary antibody dilutions were as follows: donkey anti-goat (1:400; #AB175704, Alexa Fluor 568, Abcam), donkey anti-mouse (1:400; #AB150105, Alexa Fluor 488, Abcam), and donkey anti-rabbit (1:400; #AB150075, Alexa Fluor 647, Abcam). The sections were then stained with Prolong Gold Antifade Reagent with DAPI (#8961, CST, USA). Fluorescent images were captured using a Leica TCS-SP8 laser scanning confocal microscope (Olympus Ltd.). To obtain a distinct colour contrast, we regulated the yellow signal of SOX17 from the 568 channel to the red signal.

To assess the colocalization of SOX17 and VASA and the trends of mitosis and meiosis in SOX17 positive cells, Leica TCS-SP8 counting frames were used to count the number of germ cells with positive signals. Four frames were entirely counted per section in all cases for each time point. Counting was performed by three independent observers who were unaware of the gestational age at the time-point of the investigation, and the numbers were reviewed by ImageJ software. The results are expressed as the mean value of the absolute number for each time point.

### 2.4 Statistical analysis

Statistical analysis was performed with IBM SPSS Statistics 25. Pearson correlation analysis was used for statistical analysis. Differences were considered statistically significant when *P* was less than 0.05.

## 3 Results

### 3.1 Collection and Confirmation of human female ovaries

We collected one genital ridge from multifoetal pregnancy reduction surgery and twenty-three ovaries of human foetuses (15 to 28 GW) from inevitable spontaneous abortion or induced abortion because of severe malformation of the foetus except for the urogenital system, and 3 ovarian specimens of adults undergoing ovariectomy due to autogenous diseases. We applied copy number variation sequencing (CNV-seq) to identify the ovary collected from the 7 GW embryo that was female (**Supplementary Image 1**).

**Figure 1:**
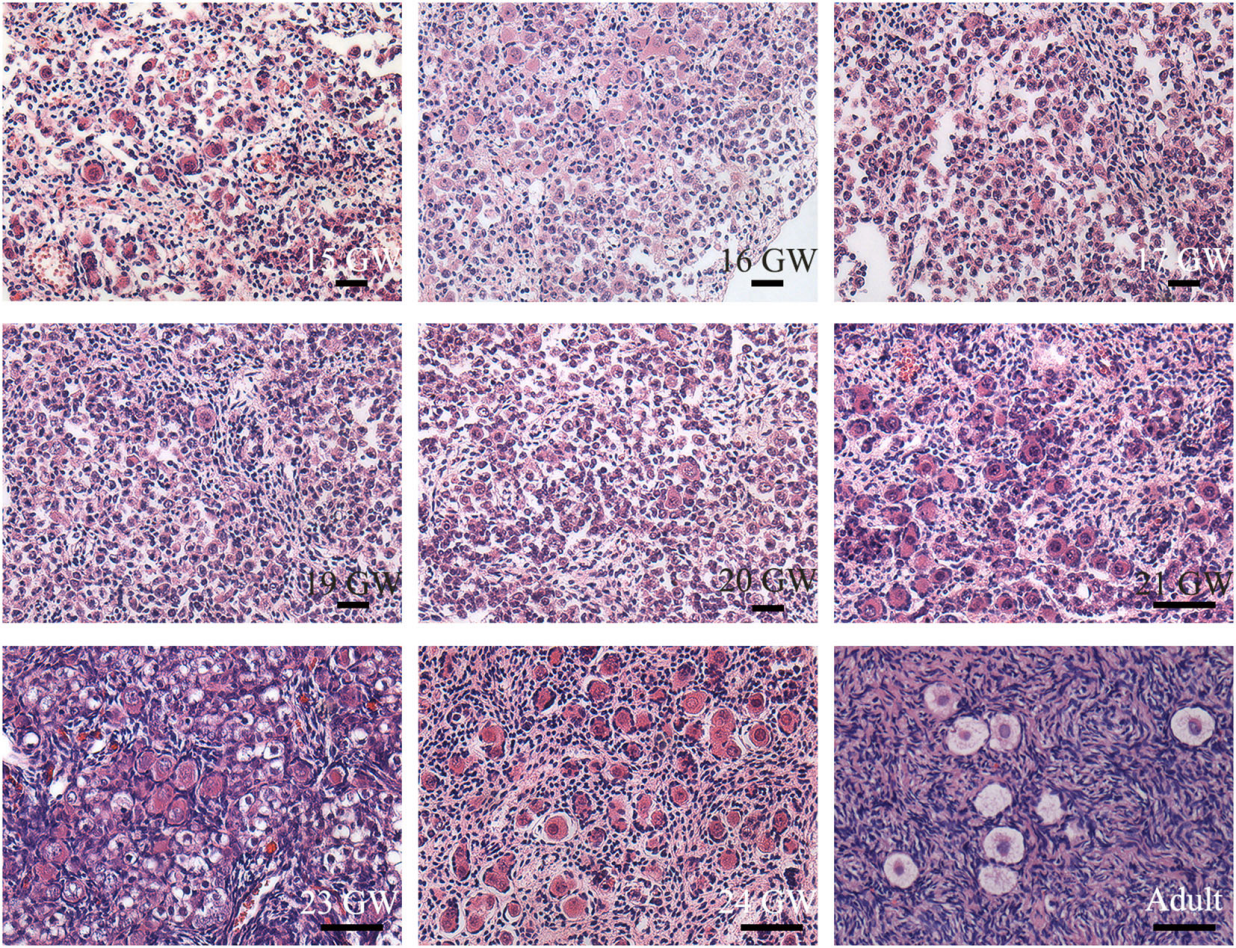
Histological sections of ovarian specimens. Representative pictures were shown. One genital ridge and ovarian specimens of foetuses (n=23) and adults (n=3) were collected. Typical oogonia (round or oval shape, chromatin was evenly distributed, and the nucleus was larger than the surrounding somatic cells) were observed in the specimens from 15 GW to 28 GW but disappeared in the adult ovary. Primordial follicles, characterized by single-layer pre−granulosa cells, were also present in ovarian sections after 15 GW. In adult ovarian sections, follicles at different stages are observed. Scale bars, 50 μm.

Typical oogonia and different developmental-stage follicles of sections were observed (**Figure 1**), confirming that the specimens collected were ovaries. Oogonia and primordial follicles were observed in sections from 15 GW. In the ovarian section from 28 GW, primary follicles were observed. In ovarian sections from adults, follicles appeared at different developmental stages, and oogonia were not observed.

### 3.2 The subcellular localization of SOX17 during the development process of human ovaries

**Figure 2:**
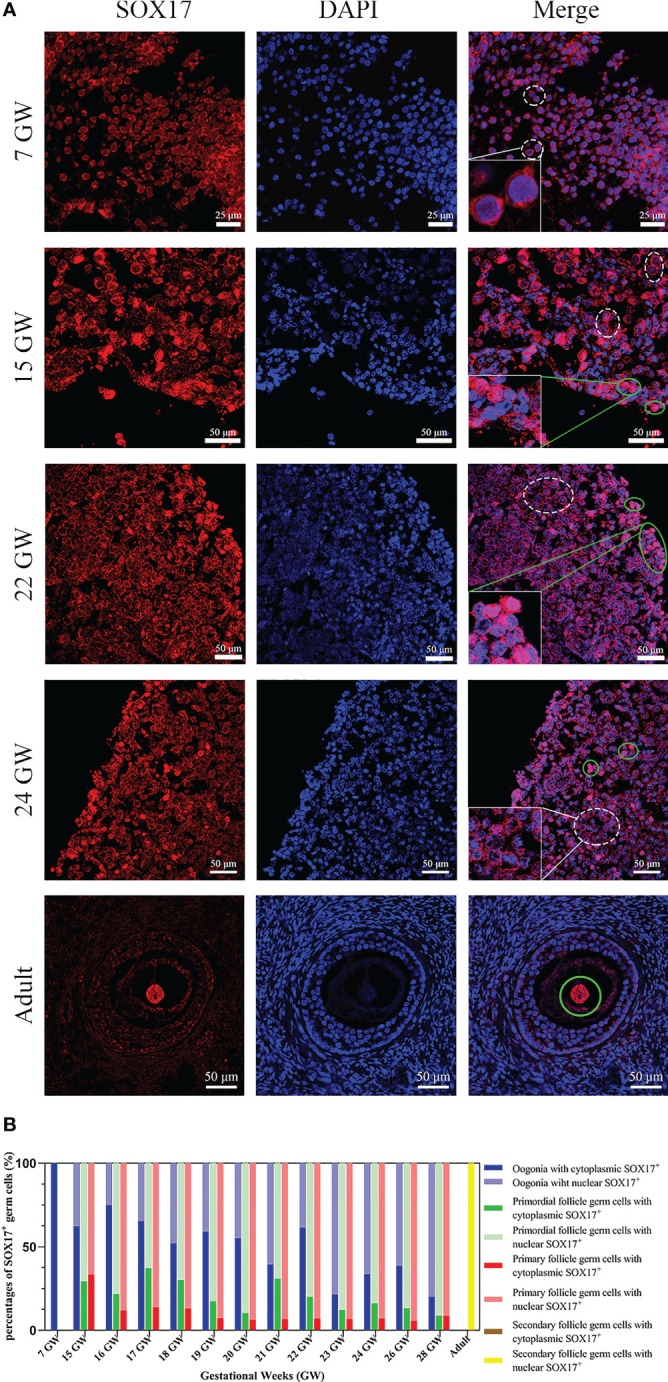
The expression pattern of SOX17 in female germ cells. **(A)** Germ cells were stained with SOX17 (red), and DAPI (blue) was used to stain the nucleus. In ovarian sections from 15 and 24 GW, SOX17 was expressed in both the cytoplasm (shown in white dashed line) and nucleus of germ cells (green full line). Signals of SOX17 were only detected in the cytoplasm of the section from 7 GW (white dashed line), and in nucleus from adult (green full line). Scale bars, 25 or 50 μm. **(B)** The percentages of germ cells (including oogonia, primordial follicles, primary follicles, and secondary follicles) with SOX17 expression in nucleus or cytoplasm were calculated. SOX17 was exclusively expressed in the cytoplasm of oogonia at 7 GW and then was detectable in both the nucleus and cytoplasm of oogonia at 15 to 24 GW, 26 GW, and 28 GW. In secondary follicles, SOX17 was only expressed in the nucleus of oocytes.

Germ cells from foetuses and adults both expressed SOX17 protein, and the subcellular localization of SOX17 was variable in different developmental stages of germ cells. SOX17 can be expressed not only in the cytoplasm but also in the nuclei. The expression of SOX17 in cytoplasm and nuclei seemed to be related to the maturation of germ cells. In the genital ridge of 7 GW, SOX17 was expressed exclusively in the cytoplasm of oogonia. SOX17 could only be detected in the oocyte nucleus of secondary follicles in adults. SOX17 expression could be detected both in cytoplasm and the nuclei of germ cells from 15 GW to 28 GW (**Figure 2A**).

For further exploration of the relationship between subcellular localization of SOX17 and the developmental stage of germ cells, the proportion of germ cells with different subcellular localizations of SOX17 was calculated (**Figure 2B**). In female germ cells, the percentages of cytoplasmic SOX17 expression showed a declining trend with the increase of gestational age, decreasing from 100% at 7 GW to 20% at 28 GW. The percentages of nuclear SOX17 expression showed an increasing trend, elevating from 0% at 7 GW to 80% at 28 GW. In addition, SOX17 was mainly localized in the nuclei of oocytes within primordial follicles (62.9%–91.2%) and primary follicles (66.7%–94.4%) (**Supplementary Table 1**).

### 3.3 Co-expression of SOX17 and VASA in the developing female genital glands

**Figure 3:**
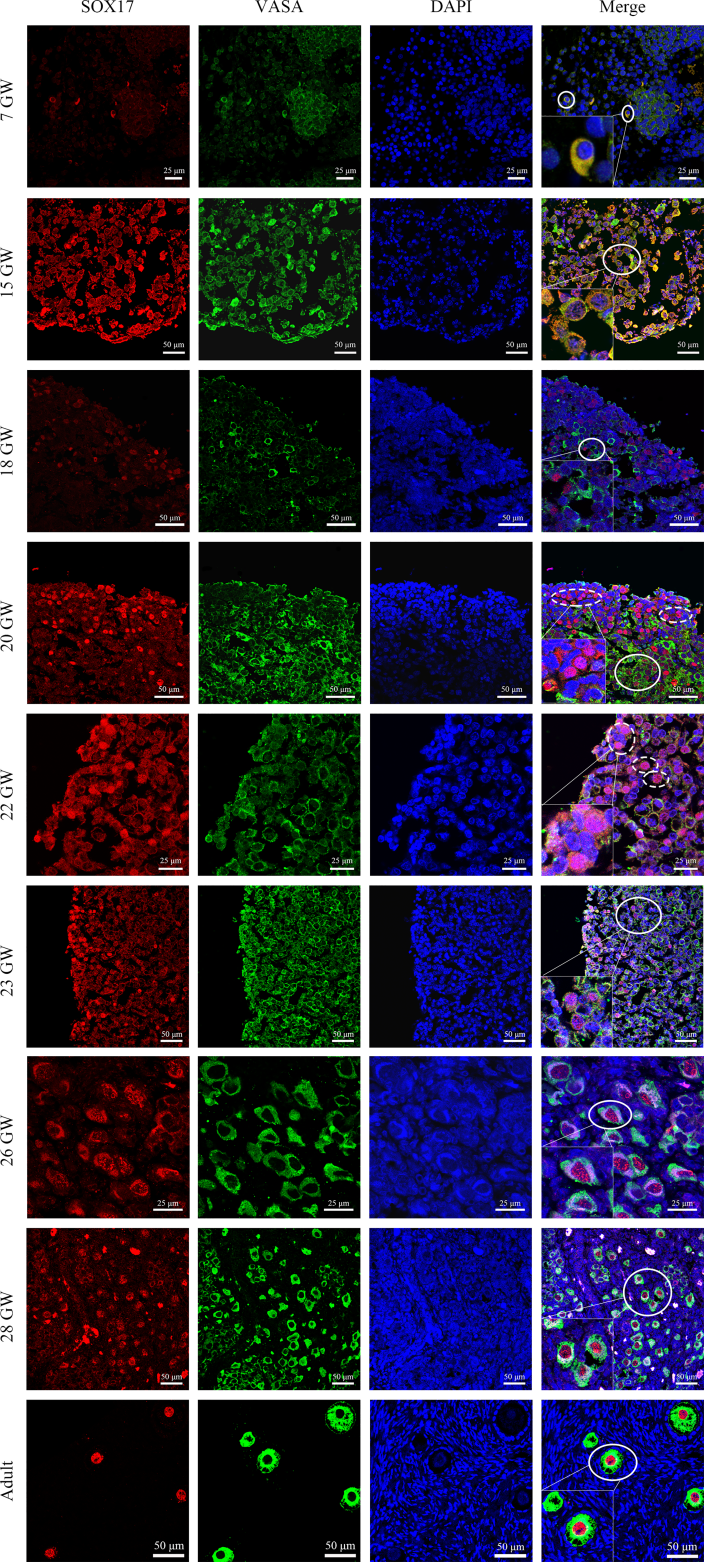
Immunolabelling of VASA and SOX17 in germ cells from genital glands during different stages. Representative pictures were shown. Germ cells were stained with SOX17 (red) and VASA (green). DAPI (blue) was used to stain the nucleus. These markers were detected in all stages of germ cells of developing human ovaries. SOX17 was expressed in a majority of germ cells marked by VASA (shown in full line). Some germ cells clustering in the ovarian cortex were positive for SOX17 (red) but not for VASA (green) (shown in dotted line). Scale bars, 25 or 50 μm.

For the purpose of investigating the co-expression pattern of SOX17 and the classic germ cell marker VASA in female germ cells during ovarian development, we co-stained human ovarian sections with antibodies of SOX17 and VASA. With the use of the double staining method, we showed the co-expression patterns of SOX17 protein and VASA protein in human female germ cells (**Figure 3**). The expression of VASA was detected in the cytoplasm of germ cells from 7 GW to adult.

**Figure 4:**
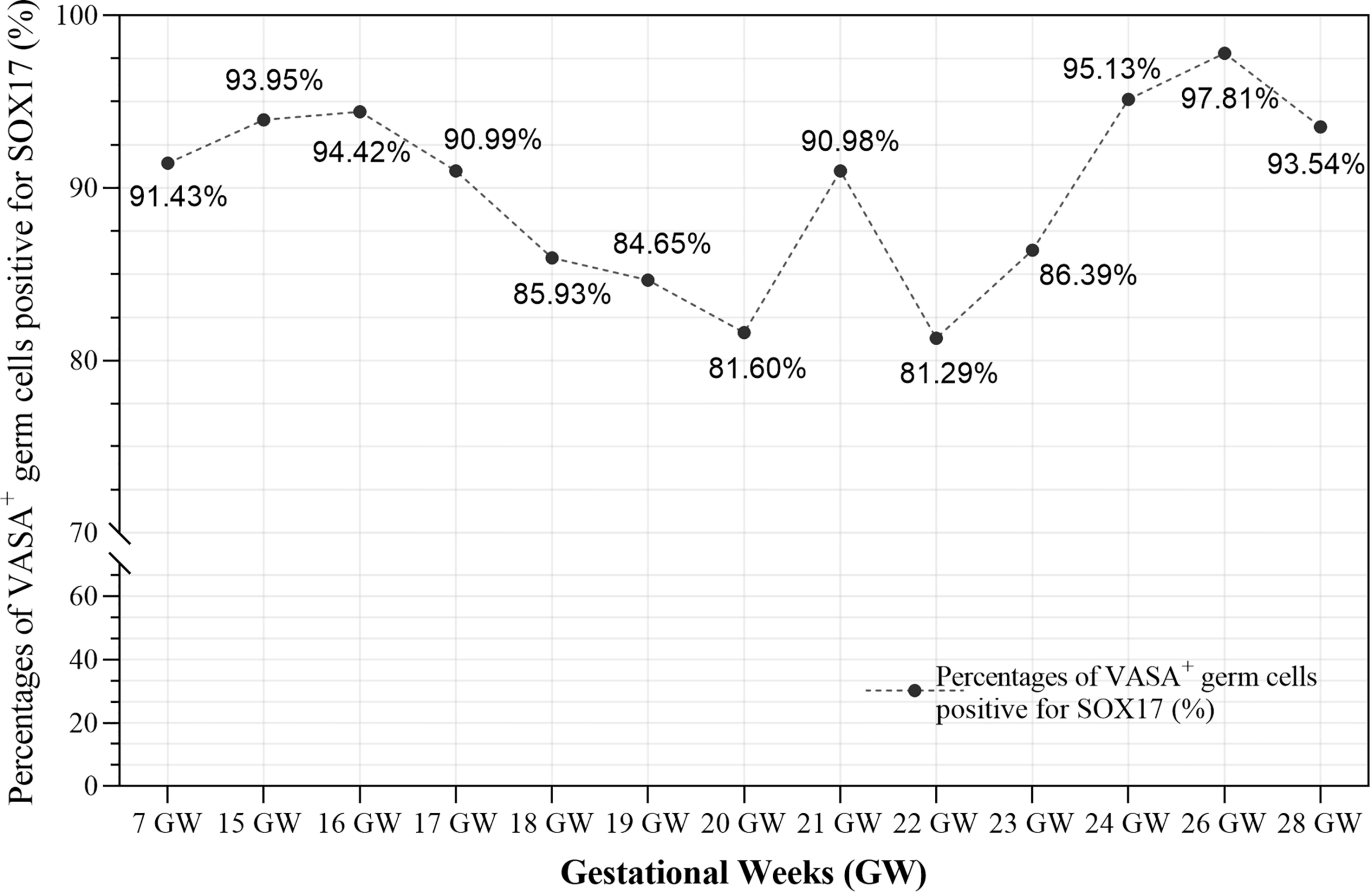
The co-expression rates of SOX17 and VASA in germ cells during different gestation ages were calculated. The rates fluctuated from 81.29-97.81% in fetal ovaries. Pearson correlation analysis showed that co-expression rates of SOX17 and VASA did not correlate with gestational weeks (r = 0.064, P = 0.835).

Furthermore, we calculated the percentages of VASA^+^ germ cells positive for SOX17 in different developmental stages (**Figure 4** and **Supplementary Table 2**). The percentage fluctuated from 81.29%–97.81% in foetal ovaries (**Figure 4**). Pearson correlation analysis showed that co-expression rates of SOX17 and VASA in germ cells did not correlate with gestational weeks (r = 0.064, *P* = 0.835).

Interestingly, we observed a subpopulation of germ cells that are immunopositive for SOX17 but not for VASA, clustering in the cortex of foetal ovaries (**Figure 3**). In these SOX17^+^VASA^-^ germ cells, the SOX17 signal was only detected in the nuclei. Representative images of these germ cells were shown (**Figure 3**).

### 3.4 The percentage of mitosis or meiosis in SOX17 immunopositive germ cells

**Figure 5:**
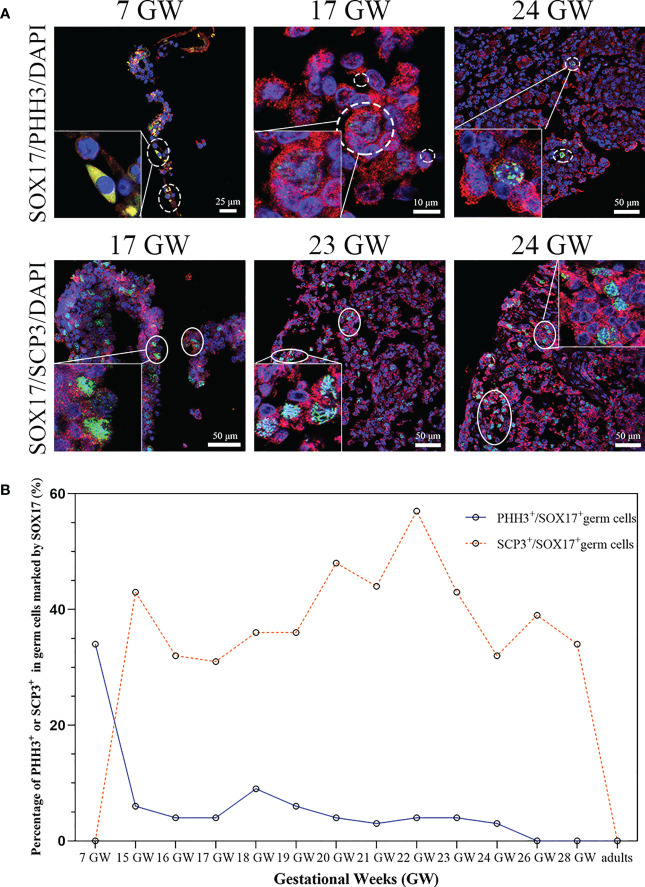
The percentage of mitosis or meiosis in SOX17 (red) immunopositive germ cells. **(A)** Representative pictures were shown. A proportion of SOX17^+^ germ cells were marked by PHH3 (shown in dashed line) or SCP3 (shown in full line). Scale bars, 10, 25, or 50 μm. **(B)** The percentages of PHH^+^ in germ cells positive for SOX17 decreased along with increasing gestational weeks. The percentage of germ cells immunopositive for both SCP3 and SOX17 was 43% in 15 GW and decreased to 0% in 28 GW and adults.

We tried to clarify the relationship between the cell division pattern and SOX17 expression in female germ cells. In SOX17^+^ germ cells, we could detect the expression of mitotic marker PHH3 or meiosis marker SCP3 independently (**Figure 5A**). PHH3 was highly expressed in SOX17^+^ germ cells at 7 GW. However, the expression percentage of PHH3 decreased significantly after 15 GW. At 7 GW, we could not observe the expression of meiotic marker SCP3 in SOX17-positive germ cells, but after 15 GW, we could see nearly 30%-50% SOX17^+^ germ cells were marked by SCP3 in foetal gonads.

We further counted the proportions of PHH3^+^SOX17^+^ germ cells and SCP3^+^SOX17^+^ germ cells (**Figure 5B**). At 7 GW, the percentage of SOX17^+^PHH3^+^ cells accounted for 34% of germ cells, but that of SOX17^+^SCP3^+^ germ cells was 0%. The proportions of SOX17^+^PHH3^+^ cells ranged from 3–9% in germ cells of 15 to 24 GW and were reduced to 0% in germ cells of 26 and 28 GW and adults. In contrast, the proportions of SOX17^+^SCP3^+^ germ cells varied from 31%–57% between 15–28 GW. In addition, PHH3 and SCP3 expression was undetectable in SOX17^+^ germ cells from adults (**Figure 5B**).

### 3.5 The co-expression features of SOX17, VASA and PHH3/SCP3 immunopositive germ cells

**Figure 6:**
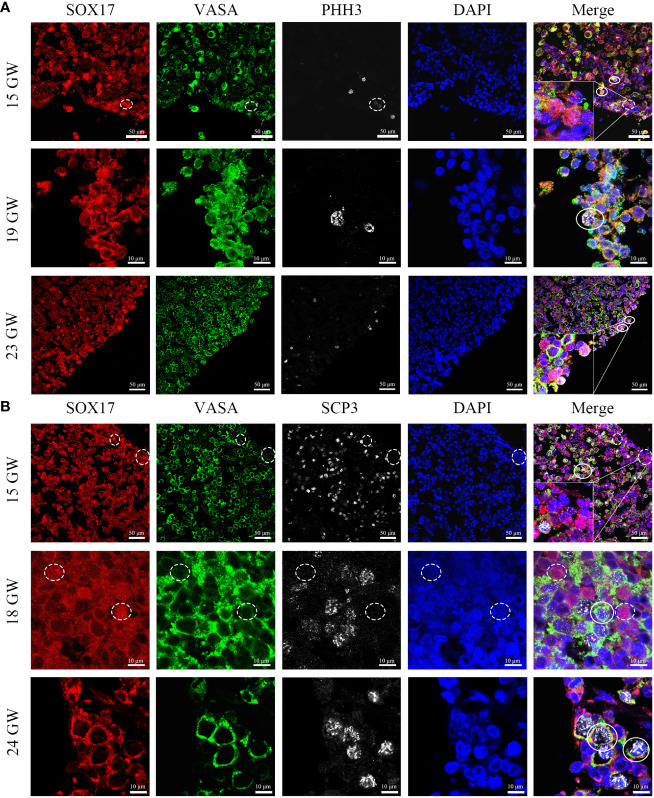
Coexpression pattern of SOX17 (red), VASA (green), and PHH3/SCP3 (silver grey) immunopositive germ cells. Representative pictures were shown. **(A)** In 15, 19 and 23 GW ovarian sections, SOX17^+^VASA^+^ germ cells were co-stained with PHH3 (white full line). In the cortex of the ovary, a small proportion of SOX17^+^VASA^-^ germ cells were also co-stained with PHH3 (white dashed line). **(B)** In 15, 18, and 24 GW ovarian sections, SOX17^+^VASA^+^ germ cells were co-stained with SCP3 (white full line). SOX17^+^VASA^-^ germ cells were not co-stained with SCP3 (shown in white dashed line). The nuclei were stained by DAPI (blue). Scale bars, 10 or 50 μm.

In order to figure out whether the co-expression status of SOX17 and VASA is related to mitosis and meiosis in female germ cells, we further co-stained germ cells with SOX17, VASA and mitosis marker PHH3 or meiosis marker SCP3 (**Figure 6**). We discovered that after 15GW, SOX17^+^VASA^+^ germ cells could appear mitosis state (PHH3^+^) and meiosis states (SCP3^+^). In addition, we detected a special population of SOX17^+^VASA^-^ germ cells also showing mitosis status (PHH3^+^) but not meiosis status (SCP3^-^). In SOX17^+^ germ cells from 15 GW to 23 GW, approximately 1.03% of these cells were positive for PHH3 but negative for VASA.

## 4 Discussion

In this study, we showed that SOX17 was detectable in germ cells from the gonadal ridge of the embryo, the ovaries of foetuses and adults. We further described that the subcellular expression of SOX17 was dynamic in germ cells at different developmental stages. We determined that there is a subpopulation of germ cells positive for SOX17 in the nucleus but not for VASA which have the potential for mitosis. These findings provide valuable insights for studies of SOX17 and human germ cells.

Müllerian ducts, which are regulated by homeobox genes during differentiation (29), and the ovary together comprise the reproductive system. As the earliest marker of hPGCLCs and a key regulator of hPGCLC fate, SOX17 attracts the interest of researchers (12, 30). In Tang’s study (31), the DNA demethylation and chromatin reorganization of human PGCs were mainly governed by a unique transcriptome, which was established by SOX17 and BLIMP1, indicating that SOX17 is critical in the DNA demethylation of PGCs. In addition, Guo et al. analysed the transcriptome and DNA methylome of human PGCs and neighbouring somatic cells from embryos between 4 and 19 GW by a single-cell RNA sequencing method (32). They reported the expression of SOX17 in human germ cells at 4, 8, 10, 11, and 17 GW at the RNA level, but the localization of SOX17 in germ cells has not yet been clarified.

Herein, we described the unique dynamic expression pattern of SOX17 in female germ cells in different developmental stages. In 7 GW, SOX17 was only expressed in the cytoplasm of germ cells. With the increase of gestational weeks, the proportion of SOX17 cytoplasmic expression in human germ cells gradually decreased, but the proportion of SOX17 nuclear expression gradually increased. It indicates that SOX17 is gradually transferred from cytoplasm to nucleus with the development and maturity of germ cells. Our study also discovered nearly 60-90% of oocytes of primordial and primary follicles in foetal ovaries expressed SOX17 in the cytoplasm. In the oocytes of secondary follicles, SOX17 was expressed exclusively in the nucleus. Our study confirms for the first time that there are two expression patterns of SOX17 in human female germ cells, and that SOX17 is gradually transferred from the cytoplasm to the nucleus along with the germ cell maturation process.

SOX17 protein was expressed in both the cytoplasm and nucleus of germ cells from foetuses but translocated to the nucleus of an oocyte from secondary follicles in adults. The subcellular localization of SOX17 in germ cells has been reported before. In 1996, Kanai et al. (17) reported two isoforms of SOX17 in mouse testes. One form of SOX17, a specific DNA-binding protein, was detectable in premeiotic germ cells. It transformed into another isoform, losing its DNA-binding ability when spermatogonia entered the pachytene stage. In addition, other research showed that SOX17 exclusively localized in the nucleus of Rhesus PGCLCs (33), or was restricted to the nucleus of Cynomolgus Monkey PGCs at embryonic day 11 (34). However, we showed that there was a nucleus-to-cytoplasm transition of SOX17 in human female germ cells during gonadal differentiation. Whether the nucleus-to-cytoplasm transition of SOX17 only exists in human female germ cells still needs more research. In addition, the specific structural changes of different SOX17 subtypes in human female germ cells still need to be clarified in future studies.

VASA has been regarded as a relatively specific marker of germ cells, especially postmeiotic germ cells (35). And VASA may regulate gonadal development by alternative splicing (36). Parte et al. (37) showed expression characteristics of VASA during ovarian stem cells differentiation and found that VASA was expressed in the cytoplasm of progenitor ovarian stem cells. In this study, we observed that VASA located in the cytoplasm of labelled germ cells from an embryo, foetuses, and adults. With double staining of SOX17 and VASA, we calculated the co-expression rates of SOX17 and VASA in germ cells, which ranged from 81.29%–97.81%. In our study, we discovered a special population of SOX17^+^VASA^-^ germ cells cluster in the cortex of ovaries. Interestingly, Anderson et al. (38) reported that VASA was detected in germ cells throughout ovaries except in the cortex. They further co-stained germ cells with VASA and OCT4, a pluripotency gene marker of embryonic stem cells and germ cells (39–41). They found that some germ cells were positive for OCT4 in the nucleus but not for VASA in the cortex, similar to germ cells positive for SOX17 but not for VASA that we described. Stoop et al. pointed out that germ cells in the cortex tended to be more proliferative than those in the medulla (35). In addition, in 1986, Konishi et al. (42) observed the ultrastructure of germ cells in foetal ovary specimens by electron microscopy and described that typical germ cells at the premeiotic stage had a 10–15 micron diameter and large, round nuclei. Hence, we hypothesized that these SOX17^+^VASA^-^ germ cells might be in the early developmental phase.

To determine whether the dynamic expression of SOX17 in germ cells was related to the mitosis and meiosis of female germ cells, we conducted multilabel immunofluorescence staining of germ cells with VASA, SOX17, and PHH3 (a mitotic marker)/SCP3 (a meiosis marker). We found that the mitotic rate of SOX17^+^ germ cells was highest at 7 GW and then declined with progressing gestational weeks. In SOX17^+^ germ cells, the mitotic rate was the highest at 7 GW (34%) and decreased to 0% after 26 GW. Double-staining germ cells with SCP3 and SOX17 showed that the meiosis rate was 0% at 7 GW, and approximately 43% at 15 GW, indicating that the occurrence of germ cell meiosis was not earlier than 7 GW and no later than 15 GW. But we could not explore the time of meiosis onset in germ cells in this study.

With immunohistochemistry techniques, Fulton et al. (43) calculated the percentage of PHH3^+^ germ cells in foetal ovaries from 14 GW to 20 GW. The percentage was approximately 1%, which was comparable to our findings. We observed that the mitosis rate of germ cells in the human female genital ridge is around 35%, which provides evidence of a large amount of SOX17^+^ germ cells undergoing proliferation at 7 GW. However, the mitosis rate of SOX17^+^ germ cells remained at about 5% and declined to 0% at 26-28 GW, which was consistent with the conclusion drawn by Kurilo et al. (44) and Baker et al. (45). We also detected the first meiosis rate of germ cells in the genital ridge and ovary of 15-28 GW. We could not observe the meiosis factor SCP3 positive in SOX17^+^ germ cells from 7 GW genital ridge, though the meiosis rate increased to around 40% in SOX17^+^ germ cells population from 15GW to 28 GW. Our results indicated that the first meiosis is quite common for SOX17^+^ germ cells population at 15 GW-28 GW. Our finding is consistent with the classic theory of human germ cells development, but we further provided the detailed proportion of germ cells that underwent mitosis and meiosis during the development of the ovary in the foetus.

In adults, the rates of mitosis and meiosis were 0% in SOX17^+^ germ cells positive, indicating that germ cells undergoing mitosis or meiosis in prophase I were absent and that neo-oogenesis may not occur in adults. This conclusion was consistent with other studies (44, 46), but further studies are needed to investigate whether there are stem cells in adult ovaries undergo mitosis.

To explore the relationship between the cell division pattern and SOX17/VASA staining in germ cells, with the triple-staining method, we showed the colocalization of VASA, SOX17, and PHH3/SCP3 in germ cells from foetal ovarian specimens. The SOX17^+^VASA^-^ germ cells population was co-stained with a mitosis marker but not with meiosis marker. However, due to the scarcity of human female gonad specimens, it is unclear whether the mitosis rate and meiosis rate differ between SOX17^+^VASA^+^ and SOX17^+^VASA^-^ germ cell population and further validation is needed.

In conclusion, we first described the relatively continuous and dynamic expression of SOX17 in different stages of germ cells from human foetuses and adults and investigated the relationship between the SOX17 dynamic expression and germ cell maturation during the development of human ovaries. The dynamic expression of SOX17 was detected in human female germ cells. Cytoplasmic SOX17 transferred into the nucleus during the process of germ cell maturation. We found that the co-expression rate of SOX17 and VASA was relatively high, while a small portion of germ cells positive for SOX17 but not for VASA was found gathering around the ovarian cortex. We described the detailed rate of mitosis and meiosis in the SOX17^+^ germ cell subpopulation. And we found out SOX17^+^ VASA^-^ germ cells subpopulation was co-stained with mitosis marker but not with meiosis marker, although we could not confirm whether mitosis rate and meiosis rate differ between SOX17^+^ VASA^+^ and SOX17^+^ VASA^-^ germ cell populations. This is the first study to depict the expression pattern of SOX17 in human germ cells of foetal and adult genital organs. This study explores the relationship between SOX17 and the proliferation and differentiation of human germ cells, to provide a theoretical basis for the treatment of patients suffering from premature ovarian failure and patients with cancer needing ovarian fertility preservation (47).

The limitations of this observational study were as follows. Due to the scarcity of human female gonad specimens, the expression of SOX17 in germ cells from 8 GW to 14 GW was deficient. The molecular mechanism of SOX17 involved in germ cell maturation during foetal ovarian development needs to be explored in future studies.

## Data availability statement

The original contributions presented in the study are included in the article/**Supplementary Material**. Further inquiries can be directed to the corresponding authors.

## Ethics statement

The studies involving human participants were reviewed and approved by the Clinical Research Ethics Committee of the First Affiliated Hospital of Sun Yat-sen University. Written informed consent to participate in this study was provided by the participants’ legal guardian/next of kin.

## Author contributions

Q-YM and C-QZ designed the study and revised this paper. Y-YL drafted this manuscript. H-YJ and M-YL contributed discussion and helped with manuscript revision. Y-YL and K-JH collected study samples. Y-YL, H-YJ, BC and XZ analyzed the data. All authors contributed to the article and approved the submitted version.
