# Supplementary figures and images for: The dynamic expression of SOX17 in germ cells from human female foetus and adult ovaries after specification
Source: Front Endocrinol (Lausanne). 2023 Jul 28;14:1124143. doi: 10.3389/fendo.2023.1124143 (PMC10422046; doi:10.3389/fendo.2023.1124143)

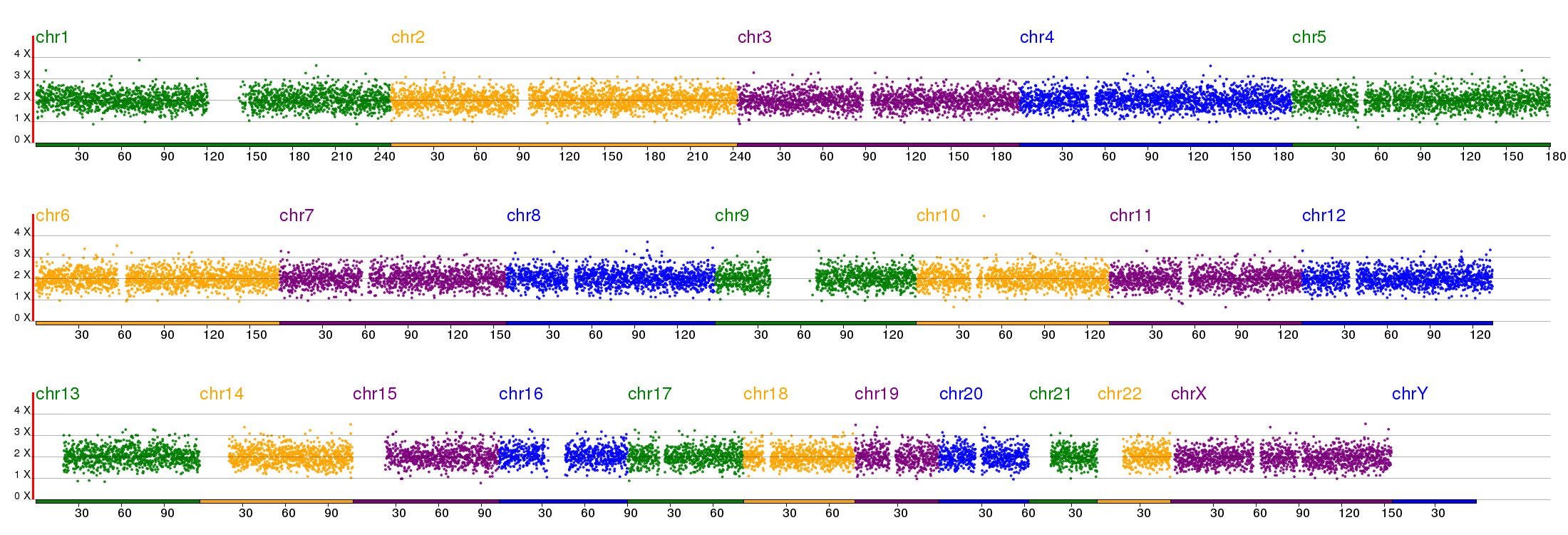

Supplement: Supplementary file 1 [file Image_1.tif]
