# Supplementary material for: The dynamic expression of SOX17 in germ cells from human female foetus and adult ovaries after specification
Source: Front Endocrinol (Lausanne). 2023 Jul 28;14:1124143. doi: 10.3389/fendo.2023.1124143 (PMC10422046; doi:10.3389/fendo.2023.1124143)
Supplement: Supplementary file 2 [file Table_1.docx]

**Supplementary Table 1.** The cellular localization of SOX17 of human female germ cells during different development stage

|  | Oogonia | | Primordial Follicle | | | Primary Follicle | | | | Secondary Follicle | |
| --- | --- | --- | --- | --- | --- | --- | --- | --- | --- | --- | --- |
|  | Cytoplasmic SOX17^+^ (%) | Nuclear SOX17^+^ (%) | | Cytoplasmic SOX17^+^ (%) | Nuclear SOX17^+^ (%) | | Cytoplasmic SOX17^+^ (%) | Nuclear SOX17^+^ (%) | Cytoplasmic SOX17^+^ (%) | | Nuclear SOX17^+^ (%) |
| 7 GW | 100.0 | 0.0 | | / | / | | / | / | / | | / |
| 15 GW | 62.2 | 37.8 | | 29.3 | 70.7 | | 33.3 | 66.7 | / | | / |
| 16 GW | 75.0 | 25.0 | | 21.6 | 78.4 | | 11.8 | 88.2 | / | | / |
| 17 GW | 65.3 | 34.7 | | 37.1 | 62.9 | | 13.8 | 86.2 | / | | / |
| 18 GW | 52.0 | 48.0 | | 30.1 | 69.9 | | 13.0 | 87.0 | / | | / |
| 19 GW | 59.0 | 41.0 | | 17.4 | 82.6 | | 7.2 | 92.8 | / | | / |
| 20 GW | 55.1 | 44.9 | | 10.3 | 89.7 | | 6.2 | 93.8 | / | | / |
| 21 GW | 39.3 | 60.7 | | 30.9 | 69.1 | | 6.7 | 93.3 | / | | / |
| 22 GW | 61.4 | 38.6 | | 20.0 | 80.0 | | 7.0 | 93.0 | / | | / |
| 23 GW | 21.4 | 78.6 | | 12.2 | 87.8 | | 6.7 | 93.3 | / | | / |
| 24 GW | 33.5 | 66.5 | | 16.1 | 83.9 | | 7.1 | 92.9 | / | | / |
| 26 GW | 38.6 | 61.4 | | 13.2 | 86.8 | | 5.6 | 94.4 | / | | / |
| 28 GW | 20.0 | 80.0 | | 8.8 | 91.2 | | 8.7 | 91.3 | / | | / |
| Adult | / | / | | / | / | | / | / | 0 | | 100.0 |
