# Supplementary material for: The dynamic expression of SOX17 in germ cells from human female foetus and adult ovaries after specification
Source: Front Endocrinol (Lausanne). 2023 Jul 28;14:1124143. doi: 10.3389/fendo.2023.1124143 (PMC10422046; doi:10.3389/fendo.2023.1124143)
Supplement: Supplementary file 3 [file Table_2.docx]

**Supplementary Table 2**. The co-expression rates of SOX17 and VASA in germ cells at different stages

| stages | ovarian specimens (n) | VASA^+^  germ cells (n) | SOX17^+^  germ cells (n) | | | SOX17^+^/  VASA^+^ germ cells (%) |  |
| --- | --- | --- | --- | --- | --- | --- | --- |
| 7 GW | 1 | 70 | | 64 | 91.43% | | |
| 15 GW | 1 | 810 | | 761 | 93.95% | | |
| 16 GW | 2 | 197 | | 186 | 94.42% | | |
| 17 GW | 1 | 222 | | 202 | 90.99% | | |
| 18 GW | 2 | 263 | | 226 | 85.93% | | |
| 19 GW | 3 | 873 | | 739 | 84.65% | | |
| 20 GW | 3 | 848 | | 692 | 81.60% | | |
| 21 GW | 2 | 543 | | 494 | 90.98% | | |
| 22 GW | 3 | 326 | | 265 | 81.29% | | |
| 23 GW | 2 | 786 | | 679 | 86.39% | | |
| 24 GW | 2 | 821 | | 781 | 95.13% | | |
| 26 GW | 1 | 731 | | 715 | 97.81% | | |
| 28 GW | 1 | 356 | | 333 | 93.54% | | |
| 24 years old | 1 | 26 | | 24 | 92.30% | | |
| 29 years old | 1 | 44 | | 41 | 93.20% | | |
| 40 years old | 1 | 13 | | 11 | 84.62% | | |
